# A221 CONGENITAL PORTOSYSTEMIC SHUNTS: FROM PRENATAL DIAGNOSIS TO LONG- TERM POSTNATAL OUTCOME

**DOI:** 10.1093/jcag/gwab049.220

**Published:** 2022-02-21

**Authors:** O Steg Saban, T Weissbach, R Achiron, M Pekar Zlotin, Y Haberman, E Kassif, B Weiss

**Affiliations:** 1 The Hospital for Sick Children, Toronto, ON, Canada; 2 Sheba Medical Center, Tel Hashomer, Tel Aviv, Israel

## Abstract

**Background:**

Congenital portosystemic shunts (CPSS) are rare vascular malformations that result in blood bypassing the liver and re-entering the systemic circulation unfiltered. The outcomes of CPSS diagnosed prenatally are unknown. Previously published data is related to patients diagnosed after symptom appearance, including hepatic encephalopathy, liver tumors and portal hypertension.

**Aims:**

To describe a cohort of prenatally diagnosed children with CPSS and report on the natural history and outcomes.

**Methods:**

The study was conducted between 2006 and 2019. Prenatal diagnosis was performed during routine prenatal ultrasound. Patients were followed by the pediatric gastroenterologists’ unit.

**Results:**

32 patients were identified; 28 patients with intrahepatic shunts and four patients with extrahepatic shunts. During follow up, Failure to Thrive (FTT) was observed in three of the patients with one shunt (16.7%), and five of the patients with two shunts or more (55.6%). The difference is significant (p- value=0.037). 24 patients with intrahepatic shunts had their shunts closed spontaneously. Median time for closure of the shunt was seven months (IQR 2–14 months, range 0–35 months). No predicting factor was detected for closure of the shunt before the age of one year. All extrahepatic shunts required surgical closure.

**Conclusions:**

Congenital intrahepatic shunts usually close spontaneously and do not need intervention. All patients in our prenatally diagnosed cohort survived with limited to no sequelae.

**Funding Agencies:**

None

